# Female homicidal strangulation in urban South Africa

**DOI:** 10.1186/1471-2458-8-363

**Published:** 2008-10-21

**Authors:** Shahnaaz Suffla, Ashley Van Niekerk, Najuwa Arendse

**Affiliations:** 1 MRC-UNISA Crime, Violence and Injury Lead Programme, P.O. Box 19070, Tygerberg, 7505, South Africa

## Abstract

**Background:**

Female strangulation in South Africa occurs in a context of pervasive and often extreme violence perpetrated against women, and therefore represents a major public health, social and human rights concern. South African studies that provide accurate descriptions of the occurrence of strangulation incidents among female homicide victims are limited. The current study describes the extent, distribution and patterns of homicidal strangulation of women in the four largest South African metropolitan centres, Tshwane/Pretoria, Johannesburg, Cape Town and Ethekwini/Durban.

**Methods:**

The study is a register-based cross sectional investigation of female homicidal strangulation, as reported in the National Injury Mortality Surveillance System for the four cities, for the period 2001 to 2005. Crude, unadjusted female strangulation rates for age and population group, and proportions of strangulation across specific circumstances of occurrence were compiled for each year and aggregated in some cases.

**Results:**

This study reports that female homicidal strangulation in urban South Africa ranges from 1.71/100 000 to 0.70/100 000. Rates have generally declined in all the cities, except Cape Town. The highest rates were reported in the over 60 and the 20 to 39 year old populations, and amongst women of mixed descent. Most strangulations occurred from the early morning hours and across typical working hours in Johannesburg and Durban, and to a lesser extent in Cape Town. Occurrences across Johannesburg, Durban and Pretoria were distributed across the days of the week; an exception was Cape Town, which reported the highest rates over the weekend. Cape Town also reported distinctly high blood alcohol content levels of strangulation victims. The seasonal variation in strangulation deaths suggested a pattern of occurrence generally spanning the period from end-winter to summer. Across cities, the predominant crime scene was linked to the domestic context, suggesting that perpetration was by an intimate partner or acquaintance.

**Conclusion:**

The study contributes to an emerging gendered homicide risk profile for a country with one of the highest homicide rates in the world. The results support the call for the development of evidence-based and gender-specific initiatives to especially address the forms of violence that instigate fatalities.

## Background

Gender-based violence persists as a global public health problem. In 2000, there were an estimated 119 000 female homicides worldwide, for an overall age-adjusted rate of 8.8 per 100 000 population [1]. Of these, the majority of deaths occurred in low- to middle-income countries, with the highest number of female homicides reported for the African Region, at a rate of 11.8 per 100 000 population. South Africa, despite its democratic transformation, the strength of its emerging economy, and widespread structural and social policy changes since 1994, is estimated to have the highest rate of intimate female homicide in the world, with 8.8 per 100 000 women age 14 years and older murdered by an intimate partner in 1999 [2].

The international body of literature on female homicide victimisation reveals a proliferation of studies during the last decade that seek to explore the patterns and correlates of homicide victimisation among women. This field of research is dominated by studies that have investigated: (1) female homicide victimisation patterns of occurrence within and between countries [3-6]; (2) the associations with social-structural factors, such as gender and economic inequality, deindustrialisation, urbanisation, ethnic heterogeneity and family disruption [7-12]; and (3) the victim-perpetrator relationship [13-16]. Studies to disaggregate female homicide victimisation, to examine the extent, distribution and patterns of occurrence of specific manners of death, such as asphyxia due to strangulation, are more limited.

Strangulation is considered to be a form of mechanical asphyxia [17]. The mechanisms of death in strangulation include airway occlusion, resulting in hypoxia; occlusion of the neck vessels or compression of the carotid arteries, leading to cerebral ischemia; and carotid sinus reflex, leading to cardiac arrest [17]. The term strangulation is specifically used to indicate the external pressure applied to the neck either by means of a ligature or the hands [17]. Descriptions of homicide victimisation by strangulation are largely to be found within the legal and forensic medicine literature. This research has focused on post-mortem accounts [18,19]; assessments of survived strangulation and mitigation of risk [20-23]; preliminary formulations of prevention [20,23,24]; and initial descriptions of the epidemiology of homicidal asphyxia in both males and females. The latter descriptive inquiry has illuminated the epidemiological profile of homicidal strangulation in both high-income settings [25-27], as well as low- to middle-income contexts [24,28-30]. In general, these studies conclude that strangulation is one of the most common forms of violent asphyxia, and accounts for approximately 10–20% of all homicide deaths in a range of countries [31], thereby representing a notable cause of death in homicide victims.

Almost all attempted or completed homicides by strangulation involve either ligature strangulation (strangulation with a cord-like object) or manual strangulation (done with the hands or forearms, or standing or kneeling on the victim's throat) [32]. Ligature strangulation is reported as the more frequently recorded method of asphyxial homicide [18,28,30,33,34]. In contrast, there appears to be significant differences in homicidal asphyxia patterns between the sexes, with some investigations on strangulation deaths observing a higher female to male ratio [20,32,34-36], and others determining a clear male preponderance among victims [18,25,30]. Findings in terms of age distribution presented as varied, with investigations reporting sharp peaks in strangulation rates for 20–30 year olds, 30–40 year olds, as well as 5–12 year olds in the case of female paediatric and adolescent strangulation deaths [25,27,30,34]. In an overwhelming number of cases studied, the victim's domestic context represented the location of the crime [24-27] which, in instances of female homicidal strangulation, appeared to further support available data signifying the victim-perpetrator relationship to be primarily intimate [25-27,36].

Many of the findings reported here are based on small numbers of cases that limit the conclusions that can be drawn, and are essentially descriptive in nature and therefore do not take into account socio-political-cultural concepts considered to be central to the understanding of gender-based violence. The results are nonetheless instructive in the preliminary identification of risk profiles for homicidal asphyxia and the development of implied interventions.

Despite the burgeoning scholarship on the subjects of female homicide victimisation and of asphyxial homicide, the research evidence reviewed highlights that systematic investigations directed specifically at strangulation in the context of female homicide victimisation remain meagre. The near absence of such research in South Africa, against the backdrop of findings emerging from the single documented national study of female homicide in South Africa [2], as well as national-level data exposing the alarmingly high homicide rates in the country [37], clearly calls for methodical inquiry into homicidal strangulation in women in South Africa. In a context plagued by persistently high rates of both fatal and non-fatal violence against women, accurate descriptions of the extent and occurrence of violent incidents are required for targeting resources, developing relevant interventions, and enabling more reliable comparisons of national and global female homicide victimisation information.

The current study explores the distribution and patterns of homicidal strangulation of women in the four largest South African metropolitan centres, Tshwane/Pretoria, Johannesburg, Cape Town and Ethekwini/Durban, for the period 2001 to 2005. These cities register amongst the highest rates of homicide in the country [38]. The following three study questions are addressed, namely:

1. What is the incidence of female strangulation in four South African cities?

2. What is the distribution of female strangulation across age and population groups?

3. What are the typical circumstances of occurrence of female strangulation?

## Methods

The study is a register-based cross sectional study of female homicidal strangulation, as reported in the National Injury Mortality Surveillance System (NIMSS) for four South African cities over the period 2001 to 2005. The study utilised mortality rates for the purpose of comparability of data, a departure from retrospective analyses of forensic autopsies and related hospital records [24,27,30].

### Injury data

The current study investigated strangulation fatality data recorded by the NIMSS. The NIMSS produces and disseminates descriptive epidemiological information on deaths due to non-natural causes that, in terms of South African legislation, are subject to medico-legal investigation. The NIMSS is a collaboration between the South African Department of Health (DoH) and the Crime, Violence and Injury Lead Programme (CVI); the latter is co-directed by the Medical Research Council (MRC) and the University of South Africa (UNISA). Information for this system is collected by the police and forensic pathologists at each mortuary, and captured onsite into a computerised database by clerks and secretarial staff. The NIMSS records 21 items of information, detailing, where available, the deceased person's age, sex, population group, province, town and suburb of injury, scene of injury, apparent manner and circumstances (or external cause) of death [39]. The NIMSS classifies the primary medical cause of death using the International Classification of Disease version 9 (ICD 9) and assigns a probable manner of death code to each case. The NIMSS also reports on the presence of alcohol and other narcotic substances in the deceased through information from forensic laboratory reports. Court findings are used to assign a final manner of death code and specify the circumstances surrounding violent deaths. The NIMSS has full coverage for Tshwane/Pretoria, Johannesburg, Cape Town and Ethekwini/Durban from January 2001 to December 2005. This includes 33 290 records for homicidal fatalities, 523 of which were due to strangulation injuries in both males and females, and 320 in females. Strangulation ranked fourth amongst all homicide deaths preceded, from most common, by firearm discharge, sharp object and blunt object homicides. As a national surveillance system, the NIMSS maintains the ethical standards prescribed by the DoH, MRC and UNISA. The database from which these records are drawn is available upon application to the DoH, via the co-ordinating research agency, the CVI.

### Denominator data

At the time of the study, the South African Census 2001 description was the most comprehensive database available for the four cities. The Census 2001 database was used as a base for city denominator extrapolations for 2002 to 2005, based on the parameters and city growth rates specified by a population model developed by the Actuarial Society of South Africa [40,41]. The extrapolated city denominators were calculated for gender (females), population group and age categories.

### Data analysis

A descriptive analysis of all female homicidal strangulations was computed for each city: by age category (split according to the categories: 0–9; 10–19; 20–29; 30–39; 40–49; 50–59; and 60 years and older); and by population group (Asian, black, coloured and white). The selected age categories were consistent with those specified for injury analyses by the WHO. Crude, unadjusted mortality rates were calculated for each city for the 2001 to 2005 period using SPSS, by relating the number of cases for each year from 2001 to 2005 to the city population estimated for each year and expressed per 100 000 per annum. The proportions of female strangulation occurring over the 5 year period were computed for each city for time (6 categories); day (7 categories); month of death (twelve categories); scene of injury (17 categories); and for blood alcohol content (BAC) (3 categories).

## Results

### Incidence of homicide and strangulation

Table 1 describes homicide and strangulation rates across four major metropolitan centres of South Africa, from January 2001 to December 2005. Cape Town recorded the highest rates of overall homicide across each of the five years, from 85.44 homicides/100 000 (in 2001) to 66.65/100 000 (in 2005). Pretoria, on the other hand, reported the lowest rates of violent deaths across this period, from 32.07/100 000 (in 2001) to 22.22/100 000 (in 2005). There was a general decline in homicide rates for all four cities over this period, with the exception of slightly elevated rates for Durban in 2002, Cape Town in 2005 and Pretoria in 2004.

**Table 1:** Homicide and homicidal strangulation by sex across four South African cities, 2001–2005

	**Year**	**2001**		**2002**		**2003**		**2004**		**2005**	
		Total deaths	Rate/100,000 pop.	Total deaths	Rate/100,000 pop.	Total deaths	Rate/100,000 pop.	Total deaths	Rate/100,000 pop.	Total deaths	Rate/100,000 pop.

**Johannesburg**	**Population**	3 225 812		3 271 105		3 303 520		3 336 255		3 383 099	
	
	**Homicide**	2247	69.65	2262	69.15	1875	56.75	1547	46.36	1432	42.32
	
	**Strangulation**	31	0.96	30	0.91	28	0.84	25	0.74	26	0.76
	
	**Female Strangulation**	20	1.23	16	0.96	12	0.7	14	0.81	15	0.85

**Durban**	**Population**	3 090 122		3 121 406		3 158 907		3 189 667		3 221 960	
	
	**Homicide**	2083	67.4	2167	69.42	2078	65.78	1903	59.66	1867	57.94
	
	**Strangulation**	34	1.1	29	0.92	43	1.36	26	0.81	20	0.62
	
	**Female Strangulation**	23	1.43	13	0.8	28	1.71	19	1.15	13	0.78

**Cape Town**	**Population**	2 893 247		2 939 810		2 981 898		3 024 589		3 068 024	
	
	**Homicide**	2472	85.44	2453	83.44	2192	73.51	1820	60.17	2045	66.65
	
	**Strangulation**	29	1	28	0.95	33	1.1	29	0.95	34	1.1
	
	**Female Strangulation**	17	1.13	17	1.1	18	1.15	22	1.38	25	1.55

**Pretoria**	**Population**	1 985 983		2 013 868		2 033 824		2 053 978		2 082 817	
	
	**Homicide**	637	32.07	620	30.78	534	26.25	593	28.87	463	22.22
	
	**Strangulation**	18	0.9	17	0.84	15	0.73	16	0.77	12	0.57
	
	**Female Strangulation**	10	0.99	12	1.16	8	0.75	8	0.74	10	0.92

The overall strangulation rate was at its highest in Durban in 2003, with 1.36 deaths per 100 000 population, and at its lowest in Pretoria in 2005, at 0.57 deaths per 100 000. There was a general decline in strangulation rates between 2001 and 2005 in Pretoria (from 0.9 to 0.57 deaths/100 000) and in Johannesburg (0.96 to 0.76/100 000). In Cape Town, the homicidal strangulation rate was relatively stable, marginally increasing from 1 to 1.1 deaths/100 000 across the five years. The overall strangulation rate varied most in Durban, from 1.1/100 000 in 2001, peaking at 1.36/100 000 in 2003, and thereafter declining to 0.62/100 000 in 2005.

**Figure 1:**
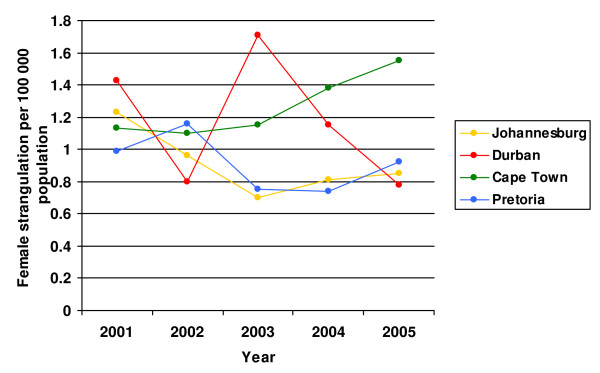
Female strangulation rates across four South African cities for the period 2001–2005.

The four major causes of homicidal death were consistently ranked across the four cities in the following order: firearm discharge, sharp objects, blunt objects and thereafter strangulation [37]. Of these, strangulation results in more female than male deaths. In general, declining rates over this period were reported for Johannesburg (from 1.23 to 0.85 deaths per 100 000) and Durban (from 1.43 to 0.78/100 000), although the Durban rates fluctuated somewhat, with a high of 1.71/100 000 in 2003 (see Figure 1). In Pretoria, rates declined from 0.99/100 000 in 2002 to 0.92/100 000 in 2005; but peaked at 1.16/100 000 in 2002. Cape Town is the one city that reported an increase, from 1.13 in 2001 to 1.55/100 000 in 2005.

### The distribution of female homicidal strangulation by age and population group

Table 2 describes the rates of female homicidal strangulation, aggregated for the five year period of the study for each city. The age of the victims was known in 82% of the cases. In three of the cities, the highest rates were reported in the over 60 year age category; in Durban (3.28/100 000), Cape Town (2.37/100 000) and Johannesburg (1.70/100 000). The highest rate for Pretoria was reported in the 20–29 year age category (1.16/100 000); with the latter category also indicating a high rate of occurrence in Cape Town (1.71/100 000). Cape Town also reported a high rate of occurrence for the 50–59 year age category (2.29/100 000).

**Table 2:** Distribution of female strangulation by age group, 2001–2005 (N = 263)

**Age group**	**Johannesburg**		**Cape Town**		**Durban**		**Pretoria**	
	Total deaths	Rate/100,000	Total deaths	Rate/100,000	Total deaths	Rate/100,000	Total deaths	Rate/100,000
**0–9**	2	0.15	3	0.23	2	0.14	1	0.12
**10–19**	4	0.32	8	0.57	10	0.64	1	0.11
**20–29**	13	0.70	26	1.71	17	1.00	12	1.16
**30–39**	13	0.84	14	1.06	22	1.72	7	0.75
**40–49**	5	0.45	11	1.07	10	1.04	2	0.30
**50–59**	6	0.94	14	2.29	5	0.85	3	0.75
**60+**	11	1.70	16	2.37	21	3.28	4	0.95

Table 3 describes female strangulation across each of the four cities by population group. In South Africa, the terms "White", "Black", "Coloured" (referring to mixed heritage) and "Asian" refer to various population groups. The use of these terms is contentious and does not imply acceptance of the racist assumptions on which these labels are based. It is recognised that these categories are a social construction that has served particular political purposes. It is not implied that such categories have any anthropological or scientific basis. The terms are used to reflect the differential manner in which the earlier South African policies of racial segregation, or *apartheid*, had impacted on the lives of various groups of South Africans, and still do. Population group affiliation was available for 98.75% of victims. Coloured females reported the highest rates of occurrence in Pretoria (3.85/100 000; n = 4), Durban (1.69/100 000; n = 4) and Cape Town (1.25/100 000; n = 47). This is followed by high rates of black female strangulations in Cape Town (1.25/100 000; n = 32) and Durban (1.15/100 000; n = 64), and by high rates amongst white women in Cape Town (1.19/100 000; n = 17) and Durban (1.15/100 000; n = 8). There were no occurrences amongst Asians in Cape Town and Pretoria where the population there constitutes a small minority [40]; rates of 1.12/100 000 were reported in Johannesburg (n = 4) and 1.09/100 000 in Durban (n = 19).

**Table 3:** Female strangulation by population group, 2001–2005 (N = 316)

**Population group**	**Johannesburg**		**Cape Town**		**Durban**		**Pretoria**	
	
	Total deaths	Rate/100,000	Total deaths	Rate/100,000	Total deaths	Rate/100,000	Total deaths	Rate/100,000
**Asian**	4	1.12	0	0	19	1.09	0	0
**Black**	54	0.88	32	1.25	64	1.15	34	0.89
**Coloured**	5	0.90	47	1.25	4	1.69	4	3.85
**White**	14	1.05	17	1.19	8	1.15	10	0.81

### Circumstances of strangulation occurrence

Table 4 describes female strangulation across each of the cities by time period; time of death was available for 72% of cases. The highest percentages of occurrence for each of the cities were reported as follows: Cape Town (29.82% between 08 h 00–11 h 59; 26.04% between 12 h 00–15 h 59); Pretoria (26.67% between 04 h 00–07 h 59; 25% in the 08 h 00–11 h 59 period); Durban (24.78% between 08 h 00–11 h 59; 23.03% between 04 h 00–7 h 59); and Johannesburg (23.51% between 04 h 00–07 h 59; 19.69% between 08 h 00–11 h 59).

**Table 4:** Occurrence of female strangulation by time period, 2001–2005 (N = 231)

**Time of death**	**Johannesburg** **% (n)**	**Cape Town** **% (n)**	**Durban** **% (n)**	**Pretoria** **% (n)**
**1 (00 h 00–03 h 59)**	10.39 (7)	15.27 (11)	12.24 (9)	3.33 (1)
**2 (04 h 00–07 h 59)**	23.51 (14)	7.08 (6)	23.03 (15)	26.67 (8)
**3 (08 h 00–11 h 59)**	19.69 (12)	29.82 (24)	24.78 (17)	25.00 (6)
**4 (12 h 00–15 h 59)**	18.24 (11)	26.04 (19)	18.43 (13)	6.67 (2)
**5 (16 h 00–19 h 59)**	11.98 (7)	14.20 (10)	15.63 (11)	15.00 (4)
**6 (20 h 00–23 h 59)**	16.19 (11)	9.02 (7)	5.88 (5)	3.33 (1)

**Figure 2:**
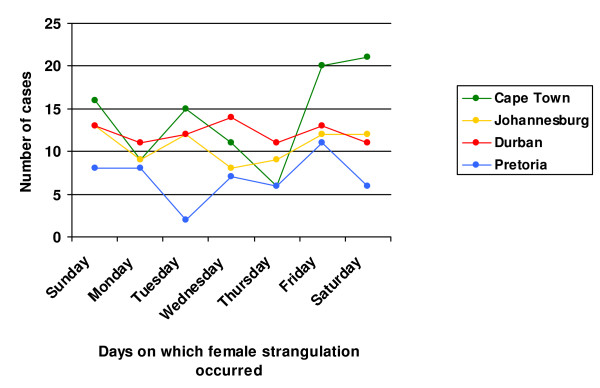
Female strangulation by day of death (N = 306).

Figure 2 describes the distribution of female strangulation by day of death, aggregated for the five year period. The day of death was known for 96% of cases. Cape Town reported its highest number of occurrences over the weekend period. Occurrences across Johannesburg, Durban and Pretoria were more evenly spread across the days of the week.

**Figure 3:**
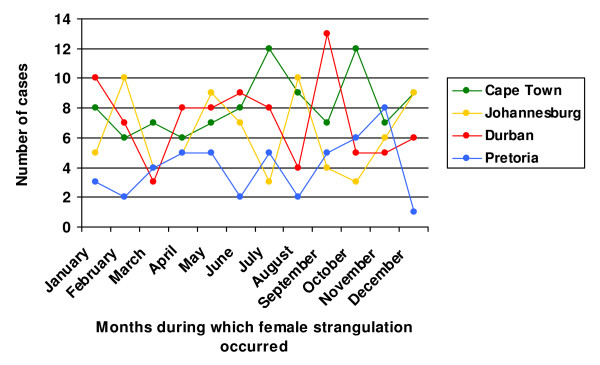
Female strangulation by month of death (N = 320).

Figure 3 describes the occurrence of cases of female strangulation for each city by month of death, for all years combined. The month of death was known for all cases. The highest rate of occurrence in each of the cities was in September for Durban (13 cases), in July and October for Cape Town (12 cases), in February and August for Johannesburg (10 cases), and in November for Pretoria (8 cases).

Across all four cities, the crime scene tends to be within the confines of the home (42% of cases in Johannesburg; 38% in Cape Town and Pretoria; and 28% in Durban). Other crime scenes included open beach or land, where 17.7% of cases occurred in Durban; residential institutes, where 14–14.6% of cases occurred in Johannesburg and Pretoria; and the road or highway, where for Cape Town 5.8% of cases occurred.

Table 5 reports on the blood alcohol content levels for selected female homicidal strangulations, representing 57% of cases. The majority of cases tested either negative for alcohol, or with a BAC within the legal limits, that is, a total of 76.5% for Johannesburg; 81.5% for Durban; and 73.3% for Pretoria. Cape Town is the only city that reported similar legal and illegal BAC levels, with 54.8% of cases testing negative and 45.2% testing above the legal BAC limit.

**Table 5:** The blood alcohol content of female homicidal strangulation cases (N = 181)

**BAC level (g/100 ml)**	**Johannesburg** **% (n)**	**Durban** **% (n)**	**Cape Town** **% (n)**	**Pretoria** **% (n)**
	
**Zero**	70.6 (36)	77.8 (21)	54.8 (40)	70 (21)
**Legal limit (< 0.05 g/100 ml)**	5.9 (3)	3.7 (1)	0 (0)	3.3 (1)
**Illegal limit (≥ 0.05 g/100 ml)**	23.5 (12)	18.5 (5)	45.2 (33)	26.7 (8)

## Discussion

This study reports that female homicidal strangulation in urban South Africa ranges from 1.71/100 000 in Durban (2003) to 0.70/100 000 in Johannesburg (2003). These rates, especially the former, are high compared to the few reported for other settings, such as the combined overall strangulation rates (for males and females) of 0.17/100 000 for Jordan and the 1.1/100 000 reported for the United States [28,42]. The South African female strangulation rates are consistent with the high overall homicide rates reported in this study for the four cities and the available national estimates, with the latter approximated to be more than seven times the global average [43]. The strangulation rates reported herein are likely to be high relative to those for other African countries, where the overall homicide rate is up to 30% lower than in South Africa [44].

Homicidal strangulation is more prominent in females than males in the metropolitan centres of the country, unlike the higher male rates observed for the other major external causes of urban homicide death, such as firearm and sharp force injury [37]. Strangulation presents as a method favoured by assailants that are considerably physically stronger than their victims, as illustrated by this higher proportion of female deaths [27]. This is also consistent with the reported association of sexual violence with strangulation [45].

Female homicidal strangulation has since 2001 generally declined in two of the cities, Pretoria and Johannesburg. Durban, despite a more erratic pattern of occurrence, also reported an overall decrease of female strangulation. This trend echoes that for the overall homicide rates across the four cities. This decrease has occurred in the context of supportive national legislation (for example, the Domestic Violence Act), the enhancement of the criminal justice system (such as the establishment of Special Offences Courts), and other social and health initiatives [46]. Despite the range of policies and interventions that have been promoted, questions have remained about the impact of these on gender-based violence, with various commentators highlighting South Africa's incomplete transition to democracy, particularly as regards the persisting indicators of gender inequity, patriarchal power, and the feminisation of poverty [47].

Cape Town is the single exception to this declining trend. The Western Cape Province (of which Cape Town is the capital and the largest metropolitan centre) also reported the highest homicide rate and amongst the highest number of reported cases of rape for this period [48]. These findings support the proposed links between sexual violence and female strangulation, as highlighted by other research [45], including investigations into sexual murders of elderly females [31]. Cape Town also reported the highest number of intoxicated strangulation victims, concordant with elevated alcohol consumption and abuse trends in the Western Cape [49], and previous reports that accentuate the association between alcohol inebriation and vulnerability to strangulation [25,27] and, more broadly, urban violence [50].

Three cities reported the highest rates of strangulation deaths amongst their older populations. Elderly female victims have been reported to be at higher risk for strangulation due to their physical vulnerability and resulting inability to offer much resistance during an attack [27,28]. The emphasis of strangulation amongst the older female population thus differs from South African reports on general female homicide (i.e. irrespective of type), which typically occurs amongst 30 to 44 year olds [44]. The next highest cluster of deaths occurs amongst young and mature adult females. This is largely consistent with other studies on strangulation, which have reported the mean age of victims to be within the 20 to 39 year range [25,27,30], and with general studies on intimate partner and non-intimate homicide, for which the average ages for South African victims are 30.4 and 41.2 years respectively [2]. This study did not reveal a significant proportion of strangulation victims to be children, unlike the findings reported in other studies [27,28], and despite the increasing proportion of child homicidal deaths in South Africa [44].

This study reported the highest rates of strangulation amongst coloured women, consistent with the indications of elevated intimate femicide rates amongst coloured females [38]. In this study, the highest rates occurred in Pretoria, Durban and Cape Town. This was followed by high rates amongst both black and white women, again in Cape Town and Durban. In general, the occurrence of high rates across these population groups in Cape Town and Durban suggests that the predisposing contexts and circumstances to strangulation may be present across, and perhaps irrespective, of population group. This study provides only initial indications of these circumstances, and the clarification of these would be the focus of further investigations into the situations that increase vulnerability to strangulation.

Most strangulations occurred from the early morning hours and across the typical working day in Johannesburg and Durban, and to a lesser extent in Cape Town. Pretoria occurrences clustered in the early morning hours until midday. These periods indicate the times of greatest vulnerability for strangulation attacks. These attacks may be directed especially at unemployed, home-based women and occur at a time of day when the majority of the population is at work or school, with consequently less immediate protection and safety. Cape Town, unlike the other cities, reported the highest rates over the weekend, consistent with high alcohol consumption over recreational periods. The seasonal variation in strangulation deaths suggests a pattern of occurrence generally spanning the period from end-winter to summer. Increasing day length has been associated with a rise in aggression in young males, and increasing evening warmth and daylight with increased consumption of alcohol in bars and streets, thereby resulting in a higher probability of inebriated encounters compared with other times of the year [26]. Across cities, the predominant crime scene is linked to the domestic context, suggesting that perpetration is by an intimate partner or acquaintance, similar to the majority of previous findings reviewed.

The study is the first of its kind in South Africa and contributes to an emerging gendered homicide risk profile for a country with one of the highest homicide rates in the world. The data is based on a well-established mandatory mortality surveillance system, which provides reliable forensic data for the cities. However, complete information on both demographics and the circumstances of cases of death are not always possible due to forensic investigative limitations. There is for example an absence of information on the victim-perpetrator relationship, which would be the focus for further South African studies on the subject. The study provides a description of strangulation victims and the circumstances of occurrence, important for the generation of overall policy and prevention guidelines. This data also lends itself to future multivariate pattern analysis, which will provide further descriptions of the scenarios of occurrence and thus more detailed prevention targets.

## Conclusion

In urban South Africa, fatal violence in females is clearly distinct from fatal violence in men. Accordingly, current prevention initiatives that address gender-based violence need to be strengthened, and supplementary evidence-based and gender-specific initiatives need to be developed to especially address the forms of violence that instigate fatalities. Dedicated policing teams; stronger legislation aimed at protecting women; universal screening for strangulation injury in women assessed to be victims of intimate partner violence; community-level substance abuse and domestic violence prevention programmes; and accessible and affordable support services and networks are some of the critical prevention initiatives that require development and support at both the local and national levels. The prevention of female homicidal strangulation is dependent on the generation of frequent, timely, comprehensive and accurate data on victims, perpetrators and the precipitating circumstances. In particular, homicidal strangulation in women needs to be further investigated in relation to intimate partner violence, as well as sex crimes. Importantly, research is required on the experiences of women who survive non-lethal strangulation to further identify risk factors. The prevention of female homicidal strangulation in South Africa will ultimately be strengthened by the creation of a social milieu that promotes equity, safety, health and human rights, and justice.

## Competing interests

The authors declare that they have no competing interests.

## Authors' contributions

This study was devised by SS and AVN. NA analysed the data. All authors made contributions to interpretation of the data. SS and AVN drafted the manuscript. All authors have read and approved the final manuscript.

## Pre-publication history

The pre-publication history for this paper can be accessed here:


